# Effect of Pantethine on Ovarian Tumor Progression and Choline Metabolism

**DOI:** 10.3389/fonc.2016.00244

**Published:** 2016-11-16

**Authors:** Marie-France Penet, Balaji Krishnamachary, Flonne Wildes, Yelena Mironchik, Delia Mezzanzanica, Franca Podo, Max de Reggi, Bouchra Gharib, Zaver M. Bhujwalla

**Affiliations:** ^1^JHU ICMIC Program, Russell H. Morgan, Division of Cancer Imaging Research, Department of Radiology and Radiological Science, The Johns Hopkins University School of Medicine, Baltimore, MD, USA; ^2^Sidney Kimmel Comprehensive Cancer Center, The Johns Hopkins University School of Medicine, Baltimore, MD, USA; ^3^Unit of Molecular Therapies, Department of Experimental Oncology and Molecular Medicine, Fondazione IRCCS Istituto Nazionale dei Tumori, Milan, Italy; ^4^Section of Molecular and Cellular Imaging, Department of Cell Biology and Neurosciences, Istituto Superiore di Sanità, Rome, Italy; ^5^Neurobiology of Cellular Interactions and Neurophysiopathology (NICN), Aix Marseille Univ, CNRS, Marseille, France

**Keywords:** choline metabolism, pantethine, ovarian cancer, orthotopic model, ascites, metastasis, high-resolution MRS

## Abstract

Epithelial ovarian cancer remains the leading cause of death from gynecologic malignancy among women in developed countries. New therapeutic strategies evaluated with relevant preclinical models are urgently needed to improve survival rates. Here, we have assessed the effect of pantethine on tumor growth and metabolism using magnetic resonance imaging and high-resolution proton magnetic resonance spectroscopy (MRS) in a model of ovarian cancer. To evaluate treatment strategies, it is important to use models that closely mimic tumor growth in humans. Therefore, we used an orthotopic model of ovarian cancer where a piece of tumor tissue, derived from an ovarian tumor xenograft, is engrafted directly onto the ovary of female mice, to maintain the tumor physiological environment. Treatment with pantethine, the precursor of vitamin B5 and active moiety of coenzyme A, was started when tumors were ~100 mm^3^ and consisted of a daily i.p. injection of 750 mg/kg in saline. Under these conditions, no side effects were observed. High-resolution ^1^H MRS was performed on treated and control tumor extracts. A dual-phase extraction method based on methanol/chloroform/water was used to obtain lipid and water-soluble fractions from the tumors. We also investigated effects on metastases and ascites formation. Pantethine treatment resulted in slower tumor progression, decreased levels of phosphocholine and phosphatidylcholine, and reduced metastases and ascites occurrence. In conclusion, pantethine represents a novel potential, well-tolerated, therapeutic tool in patients with ovarian cancer. Further *in vivo* preclinical studies are needed to confirm the beneficial role of pantethine and to better understand its mechanism of action.

## Introduction

Ovarian cancer is the leading cause of death from gynecological malignancies with an incidence of 220,000 cases worldwide per year (1). Although the prognosis in cases detected at an early stage is quite favorable, the vast majority of cases are diagnosed at an advanced stage, when 5-year survival rates are only 30–40%. Median life expectancy for ovarian cancer patients is 5 years, and about 80% of diagnosed patients will eventually succumb to it (2). The poor prognosis of epithelial ovarian cancer (EOC) is due to a combination of the aggressive characteristics of the disease and an unpredictable response to front-line therapy, further compounded by late detection of the disease and resistance of ovarian cancers to current treatments (3). The primary treatment for EOC consists of aggressive cytoreductive surgery, followed by chemotherapy with platinum and taxane (4). Although platinum and taxane combination remains the standard treatment for EOC, new drug combinations (5) as well as different administration schedules (6) are being tested and might be reasonable options for first-line treatment of women with advanced EOC. Recently, the introduction of anti-angiogenic drug combined to front-line treatment has been also proposed (7, 8). Current first-line chemotherapies for advanced diseases are listed in Table 1.

**Table 1:** **Front-line treatment for ovarian cancer patients**.

Carboplatinum + paclitaxel (1)	Platinum-based chemotherapy + anti-mitotic chemotherapy
Carboplatinum and pegylated doxorubicin (5)	Platinum-based chemotherapy + intercalating DNA chemotherapy
Carboplatinum + weekly paclitaxel (6)	Platinum-based chemotherapy + anti-mitotic chemotherapy
Carboplatinum and taxol + bevacizumab (7, 8)	Platinum-based chemotherapy + anti-mitotic chemotherapy + angiogenesis inhibitor

Metastases and malignant ascites are complications frequently observed in late-stage ovarian cancer. Intraperitoneal seeding is the most common route of dissemination (9), although direct invasion or dissemination through the lymphatics and vasculature also occur. Malignant ascites function as a permissive reactive tumor-host microenvironment and provides sustenance for floating tumor cells (10). This results in an abnormal build-up of fluid in the abdomen, causing discomfort, pain, problems with mobility and breathing, and other symptoms that decrease the quality of life. Despite the improvement of surgical approaches and drug development, EOC patients have experienced little improvement in overall survival in the last 30 years (11). New therapeutic strategies exploiting novel targets are urgently needed to minimize morbidity, improve survival rates, and to eventually cure patients.

In the present study, we applied magnetic resonance imaging (MRI) and high-resolution magnetic resonance spectroscopy (MRS) to assess the use of pantethine as a therapeutic agent in an orthotopic model of ovarian cancer. We used an orthotopic model in which the relevant tumor physiological environment is maintained and that frequently forms metastases and malignant ascites. Pantethine is the stable disulfide form of pantetheine, the precursor of vitamin B5 (pantothenic acid). As a part of the active moiety of coenzyme A (CoA), it is a key regulator in lipid metabolism (12–14). Pantethine has the advantage of being an anti-inflammatory and hypolipidemic agent with very few side effects. Pantethine has been shown to prevent the perivascular inflammation and to protect mice against the cerebral syndrome associated with malaria (15); the protection was associated with a significantly lower level of circulating tumor necrosis factor (TNF)-α (15). TNF-α has been linked to multiple steps of tumorigenesis, including cellular transformation, promotion, survival, proliferation, invasion, angiogenesis, and metastasis (16). Pantethine has also been shown to inhibit CXCL12/CXCR4-induced cell transendothelial migration (17). With its anti-inflammatory and hypolipidemic properties, pantethine appeared to be a good novel candidate against ovarian cancer progression, metastases, and ascites formation.

## Materials and Methods

### Cell Line and Tumor Implantation

NIH: OVCAR3 cells from American Type Culture Collection (ATCC, VA, USA) were used in the present study. OVCAR3 cells are human epithelial ovary adenocarcinoma cells originally isolated from a malignant effusion. Cells were cultured in RPMI 1640 (Sigma Chemical Co., St. Louis, MO, USA) with 10% fetal bovine serum (Sigma Chemical Co., St. Louis, MO, USA). Tumor implantation was performed using 6- to 8-week-old severe combined immunodeficient (SCID) female mice. We used a two-step process for orthotopic tumor implantation (Supplementary Material). We first generated subcutaneous tumors by inoculating a cell suspension of 2 × 10^6^ cells in 0.05 ml of Hanks balanced salt solution in the flank of SCID female mice. Once the tumor reached a size of 100–200 mm^3^, it was excised and cut into small pieces under sterile conditions. Orthotopic implantation was then performed by surgically transplanting a piece of tumor tissue onto the ovaries of a separate group of SCID mice. All surgical procedures and animal handling were performed in accordance with protocols approved by the Johns Hopkins University Institutional Animal Care and Use Committee and conformed to the Guide for the Care and Use of Laboratory Animals published by the NIH.

### Treatment Protocol

The treatment was started when the tumors reached about 100 mm^3^ with a daily i.p. injection of saline for the control group, and pantethine (Sigma Chemical Co., St. Louis, MO, USA) diluted in saline for the treated group (750 mg/kg).

### *In Vivo* MR Examination

Non-invasive MRI was used to assess tumor growth in deep-seated tissue using T_1_-weighted imaging and diffusion-weighted imaging. All imaging studies were performed on a 4.7-T BrukerAvance (Bruker, Billerica, MA, USA) spectrometer using a home-built volume coil placed around the torso of the anesthetized mice. Animals were anesthetized with a mixture of ketamine (6.25 mg/kg) and acepromazine (62.5 mg/kg) administered i.p. A pad circulated with warm water was used to maintain animal body temperature. Multi-slice T_1_-weighted images and multi-slice diffusion-weighted images, with an in-plane spatial resolution of 250 μm × 250 μm (128 × 128 matrix, 32 mm field of view, *b*-value of 100 mT/m), were acquired to localize the orthotopic tumors that appear hyperintense on these images.

### MR Spectroscopy of Dual Phase Extracts

High-resolution proton MRS of tumor tissue extracts was applied to assess water phase and lipid phase metabolites in tumor extracts. Lipid- and water-soluble fractions were obtained from tumors using a dual-phase extraction method with methanol/chloroform/water (1/1/1) (18). Briefly, tissues were freeze-clamped and ground to powder. Ice-cold methanol was added to the powder, and the samples were homogenized. Ice-cold chloroform, followed by ice-cold water, was added, and the samples were kept at 4°C overnight for phase separation. Samples were centrifuged for 30 min at 15,000 *g* at 4°C to separate the phases. The water/methanol phase containing the water-soluble metabolites was treated with chelex (Sigma Chemical Co., St. Louis, MO, USA) for 10 min on ice to remove divalent cations. Methanol was removed by rotary evaporation, and the remaining water phase was lyophilized and stored at −20°C. The chloroform phase containing the lipids was dried in a stream of N_2_ and stored at −20°C. Water-soluble samples were dissolved in 0.5 ml of D_2_O (Sigma Chemical Co., St. Louis, MO, USA) containing 3-(trimethylsilyl) propionic-2,2,3,3,-d4 acid (Sigma Chemical Co., St. Louis, MO, USA) as an internal concentration standard (sample pH of 7.4). Lipid samples were dissolved in 0.6 ml of CDCl_3_/CD_3_OD (2/1) containing tetramethylsilane as an internal concentration standard (CDCl_3_ and CD_3_OD premixed with tetramethylsilane by the manufacturer, Cambridge Isotope Laboratories, Inc.). Fully relaxed ^1^H MR spectra of the extracts were acquired on a BrukerAvance 500 spectrometer operating at 11.7 T (BrukerBioSpin Corp., Billerica, MA, USA) using a 5-mm HX inverse probe and the following acquisition parameters: 30° flip angle, 6,000 Hz sweep width, 12.7 s repetition time, time-domain data points of 32k, and 128 transients (18). Spectra were analyzed using the Bruker XWIN-NMR 3.5 software (BrukerBioSpin). Integrals of the metabolites of interest were determined and normalized to the tumor weight. To determine concentrations, peak integration from ^1^H spectra for all metabolites studied was compared to the internal standard.

### Metastases and Ascites

Presence of ascites was recorded at necropsy. Lymph nodes, lungs, and livers were fixed in formalin, paraffin embedded, sectioned, and stained with hematoxylin and eosin (H&E) for further analysis. The presence of metastases was checked on H&E stained sections of the lymph nodes, liver, and lungs.

### Immunohistochemistry

The 5-μm thick formalin fixed sections were used for Immunohistochemistry (IHC) analysis. Antigen retrieval was achieved by boiling sections in citrate buffer solution (pH 6) for 20 min. Sections were stained for proliferation using Ki-67 (rabbit polyclonal, Thermo Fisher, Rockford, IL, USA, 1:100 dilution), and for apoptosis using Caspase-3 (8G10, rabbit polyclonal, Cell Signaling, Danvers, MA, USA, 1:100 dilution) following standard protocols, and further processed by addition of biotinylated anti-rabbit IgG and ABC reagent (PK-4001, Vector laboratories, Burlingame, CA, USA). Detection was achieved by addition of the chromogen DAB (3, 3′-diaminobenzidine, Dako, Carpinteria, CA, USA). Images were captured by scanning the immunostained sections at high resolution on an Aperio ScanScope^®^ CS System at 20 × resolution (Leica Biosystems Inc., Buffalo Grove, IL, USA). Analysis of the slides was performed using the algorithms and protocols developed by the company.

### Toxicity Analysis

The toxicity analyses were performed in MDA-MB-231 tumor-bearing mice. The 2 × 10^6^ cells were injected orthotopically into the mammary fat pad of 6- to 8-week-old female SCID mice. The treatment was started when the tumors reached about 100 mm^3^ with a daily i.p. injection of saline for the control group and pantethine for the treated group (750 mg/kg) (*n* = 5). Mice were treated for 3 weeks and weighed once a week. At the end of the treatment period, mice were sacrificed. Kidney and liver function were evaluated from serum creatinine, blood urea nitrogen (BUN), serum alanine aminotransferase (ALT), and aspartate aminotransferase (AST) levels obtained at the Johns Hopkins University School of Medicine Phenotyping Core Facility, using spectrophotometric measurements obtained with an automated Vet Ace Clinical Chemistry system (Alfa Wasserman Diagnostic Technologies LLC, NJ, USA).

### Statistical Analysis

Values were displayed as mean ± SEM. Statistical significance was evaluated using the Student’s *t*-test; *p* < 0.05 was considered significant.

## Results

**Figure 1:**
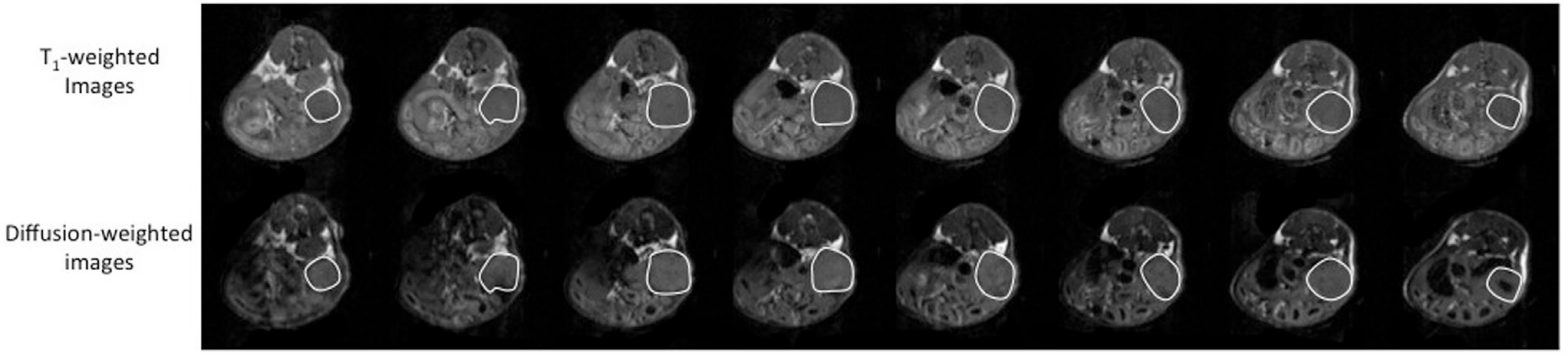
**Representative adjacent T_1_-weighted images (top row) and diffusion-weighted images (bottom row) of an orthotopically implanted OVCAR3 tumor-bearing mouse**. The tumor is highlighted by a white line. The tumor volume was measured by determining the tumor area on each 1-mm thick slice and by adding the areas to calculate the total tumor volume.

**Figure 2:**
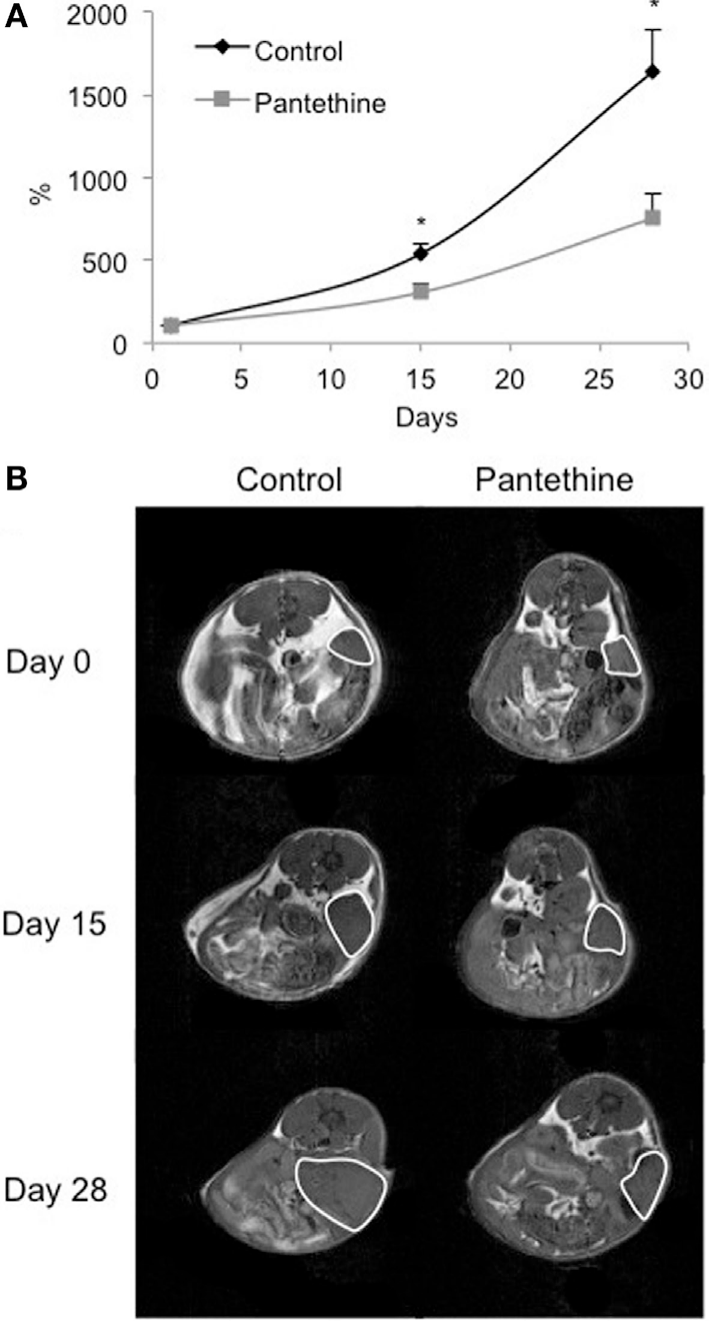
**Normalized tumor growth curves from control mice and pantethine-treated mice (A) and representative T_1_-weighted images of a central slice of tumors imaged at days 0, 15, and 28 of a control mouse (left column) and a treated mouse (right column) (B)**. *n* = 13 and 14, respectively; *P < 0.05.

To assess the efficacy of pantethine on tumor progression, metastases, and ascites formation, we used an orthotopic model of ovarian cancer. The orthotopic implantation was performed to maintain the relevant tumor physiological environment. Ovarian cancer cells are typically injected into the peritoneal cavity, inducing ascites and peritoneal spread of tumor, but most cell lines do not form solid tumors. Instead, here we performed microsurgical orthotopic implantation of ovarian cancer tissue onto the ovary of female SCID mice. In our model, ascites and metastases in the peritoneal cavity, in the liver, on the diaphragm, and in distal lymph nodes are frequent, similar to human disease. Tumor growth was measured following implantation by imaging the mice weekly with MRI (Figure 1). The treatment consisting of daily i.p. injections of pantethine at a dose of 750 mg/kg commenced when tumors were ~100 mm^3^. Under these conditions, no side effects and no significant weight loss were observed in the treated group compared to the control group (20.8 ± 0.9 versus 22.2 ± 2.8 g, respectively). The control group was injected daily with saline. Tumor growth was followed weekly non-invasively by MRI on a 4.7-T spectrometer. Tumor areas were measured for each 1-mm thick slice, and the values were added to assess the total tumor volume. We observed a significant reduction of tumor growth in the treated group compared to the control group (Figure 2).

**Figure 3:**
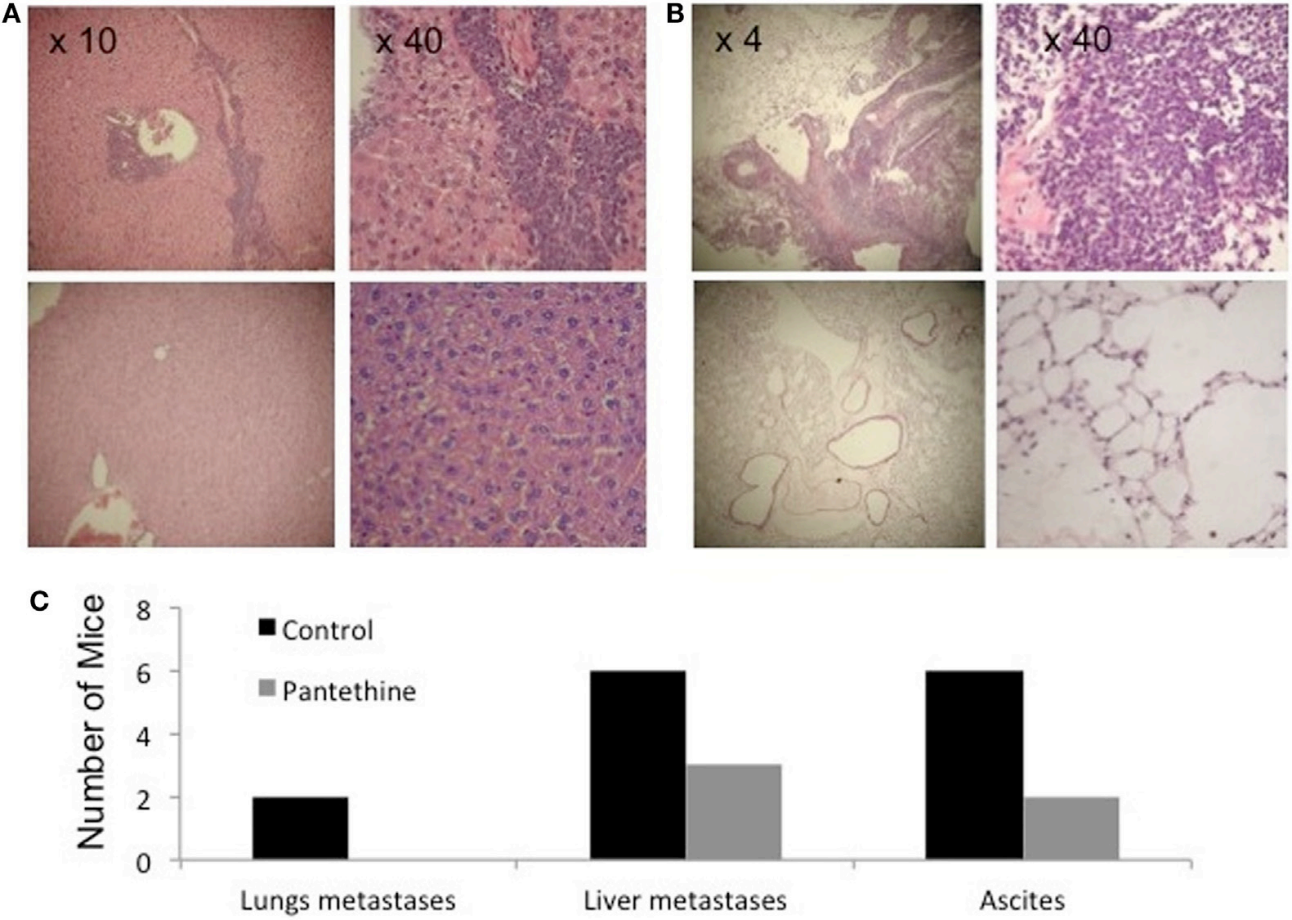
**Representative H&E stained sections of liver (A) and lungs (B) from a control mouse (top row) and a treated mouse (bottom row)**. **(C)** Histogram representing the number of control and treated mice with metastases in the lungs, in the liver, and with ascites (*n* = 7).

**Figure 4:**
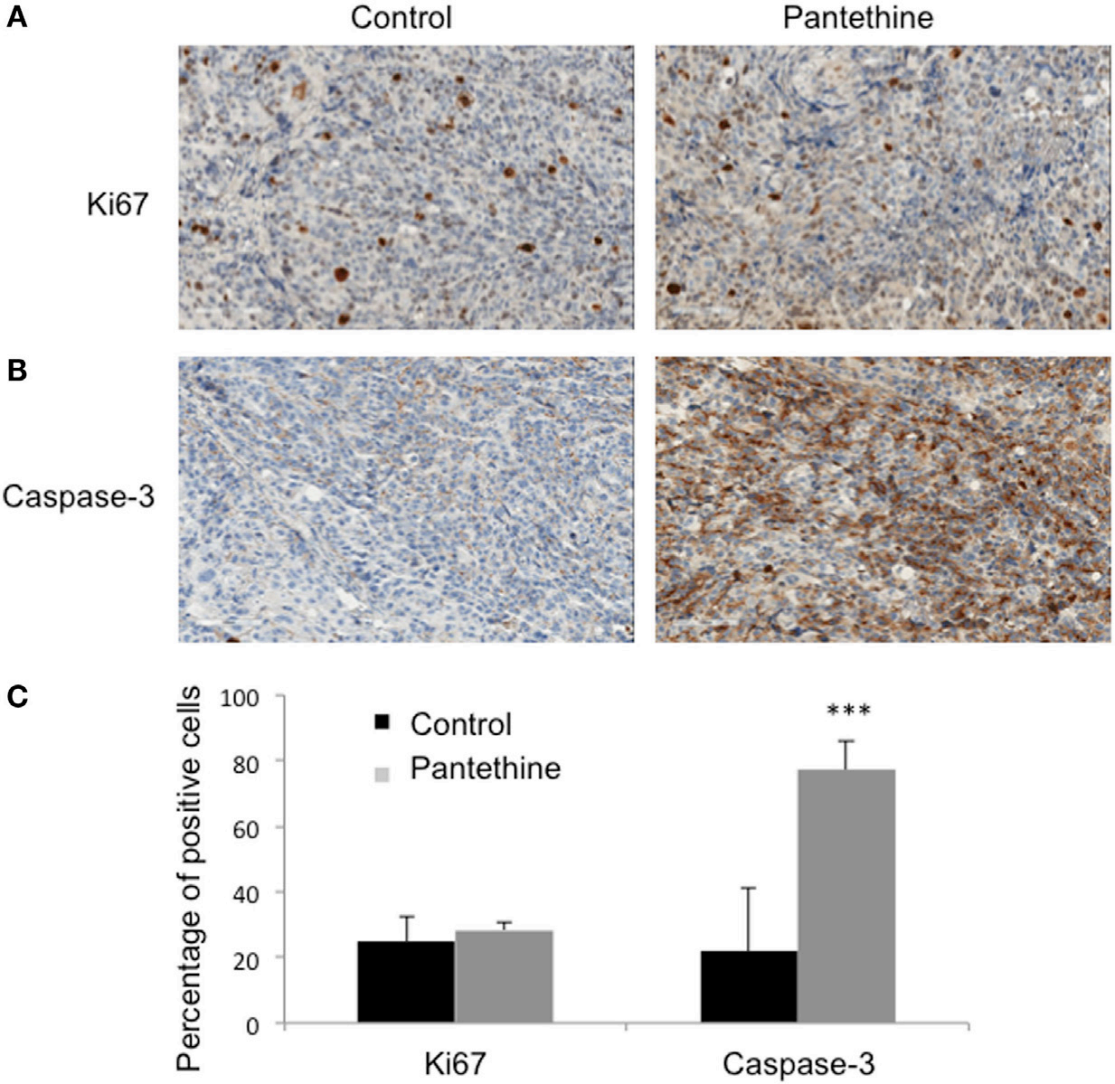
**Representative IHC stained sections of control and treated tumors for Ki-67 (A) and Caspase-3 (B)**. **(C)** Histogram representing the percentage of positive cells for each marker in control and treated tumors (*n* = 3; mean ± SD are represented; ***P < 0.001).

When we sacrificed the mice after 4 weeks of treatment, we observed liver metastases in 86% of control mice (6/7), but only in 43% of treated mice (2/7), lungs metastases in 29% of control mice (2/7), and none in treated mice and ascites in 86% of control mice (6/7), and 29% of treated mice (2/7) (Figure 3). IHC analysis of tumor sections did not show any statistically significant differences in proliferation rates (Figures 4A,C). Higher levels of caspase-3 were measured in the treated tumors compared to control tumors (Figures 4B,C).

**Figure 5:**
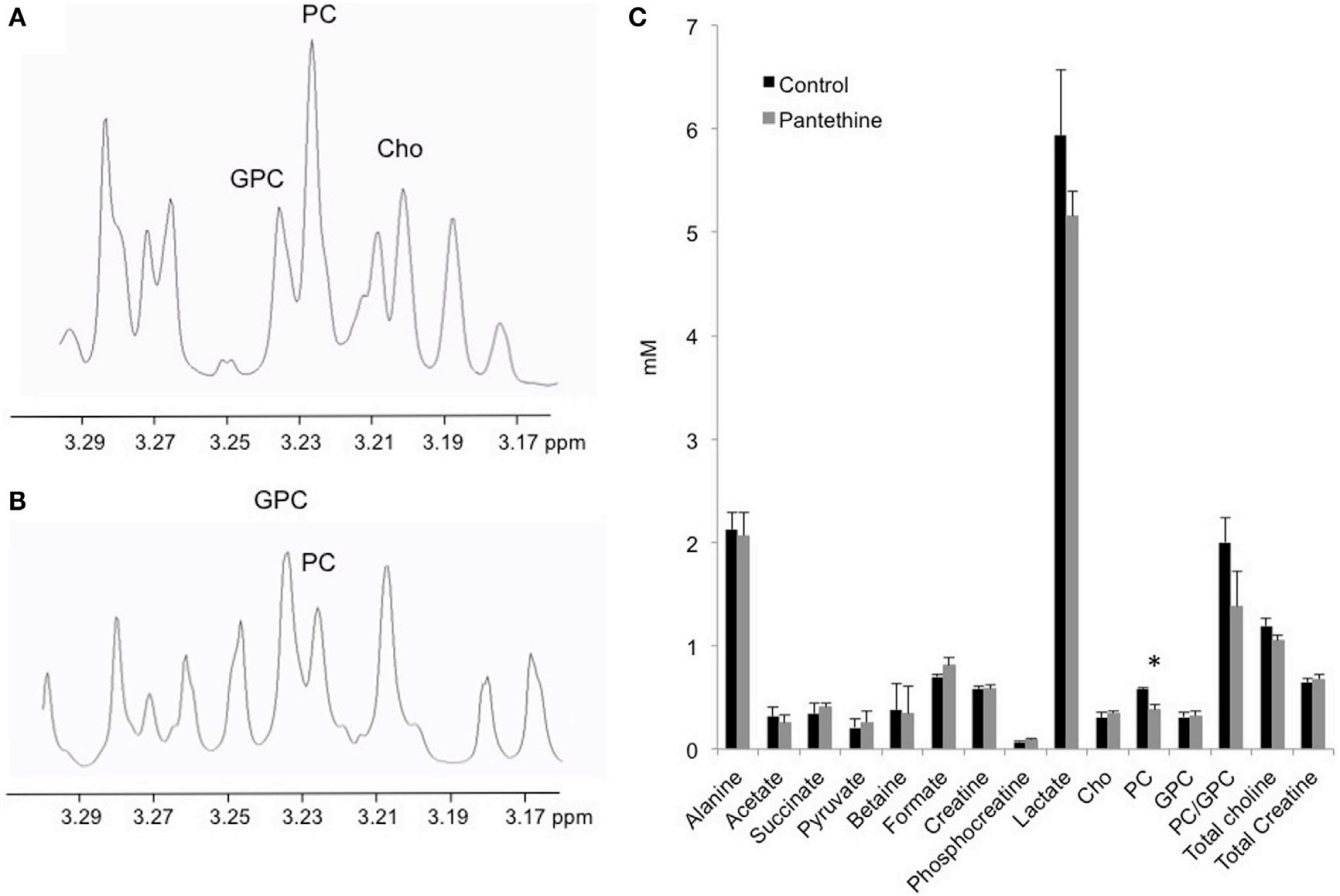
**Representative water phase ^1^H MR spectra centered around the 3.2 ppm region from a control (A) and a treated (B) mice**. **(C)** Metabolite quantification from control and treated tumor extracts (*n* = 5; mean ± SD are represented; *P < 0.05).

To assess the effect of the treatment on the tumor metabolism, we analyzed tumor extracts with high-resolution ^1^H MRS. We performed dual-phase extraction of the tumors to assess the lipid phase and the water phase. Representative water phase ^1^H MR spectra centered around the 3.2 ppm region of a control tumor and a pantethine-treated tumor are shown in Figures 5A,B, respectively. A significant decrease of phosphocholine (PC) in the treated tumors was observed (Figure 5C). No differences were observed in the other metabolites measured, including lactate.

**Figure 6:**
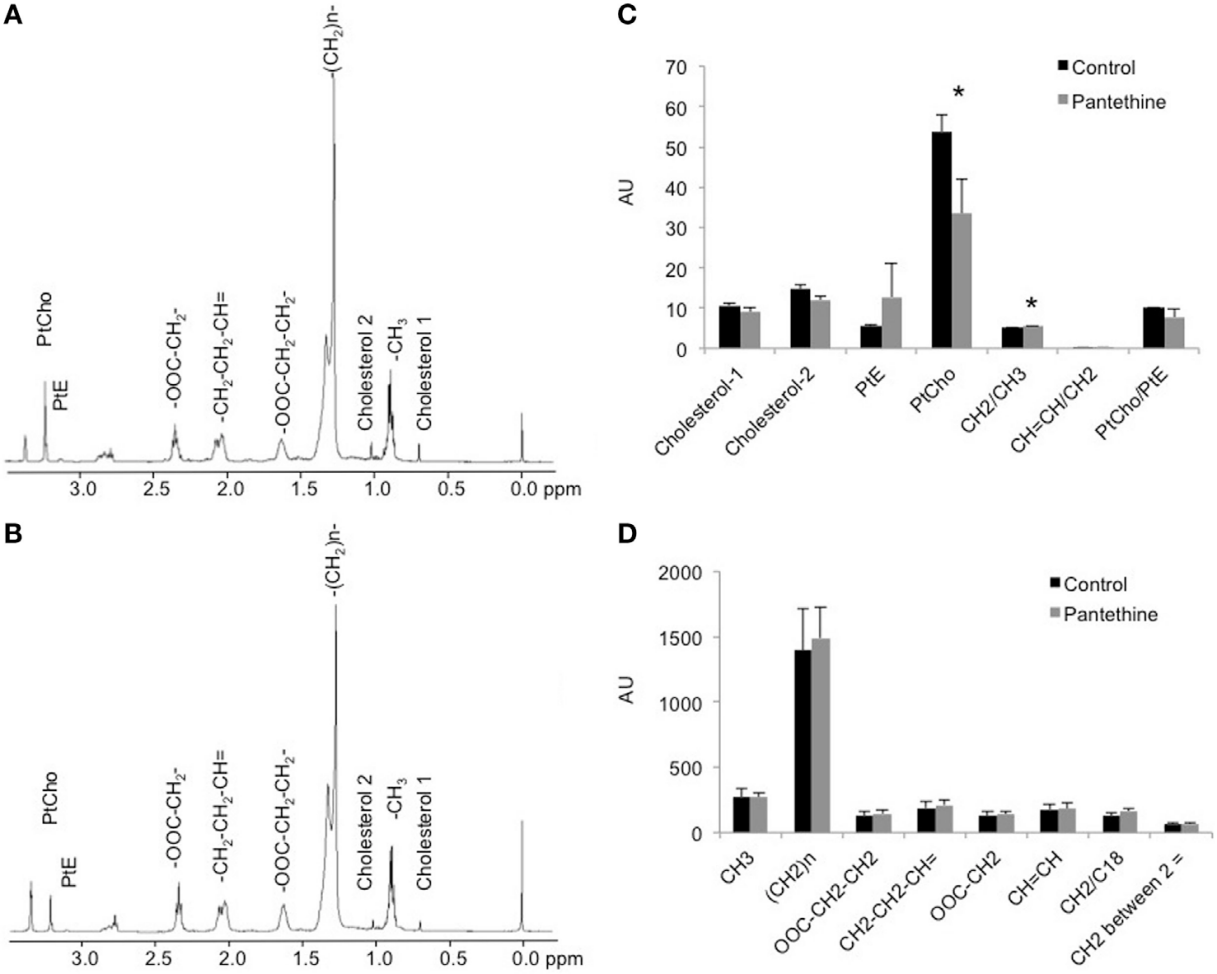
**Representative lipid phase ^1^H MR spectra from a control (A) and a treated (B) mice**. **(C,D)** Lipid quantification in arbitrary unit (AU) from control and treated tumor extracts (*n* = 5; *P < 0.05).

We next measured the lipid concentration in the lipid phase. Representative spectra of a control tumor and a treated tumor are shown in Figures 6A,B, respectively. Analysis of the spectra revealed a significant decrease of phosphatidylcholine (PtCho) in the pantethine-treated tumors compared to the controls (Figure 6C). No differences were observed in the other lipids assessed (Figures 6C,D).

**Figure 7:**
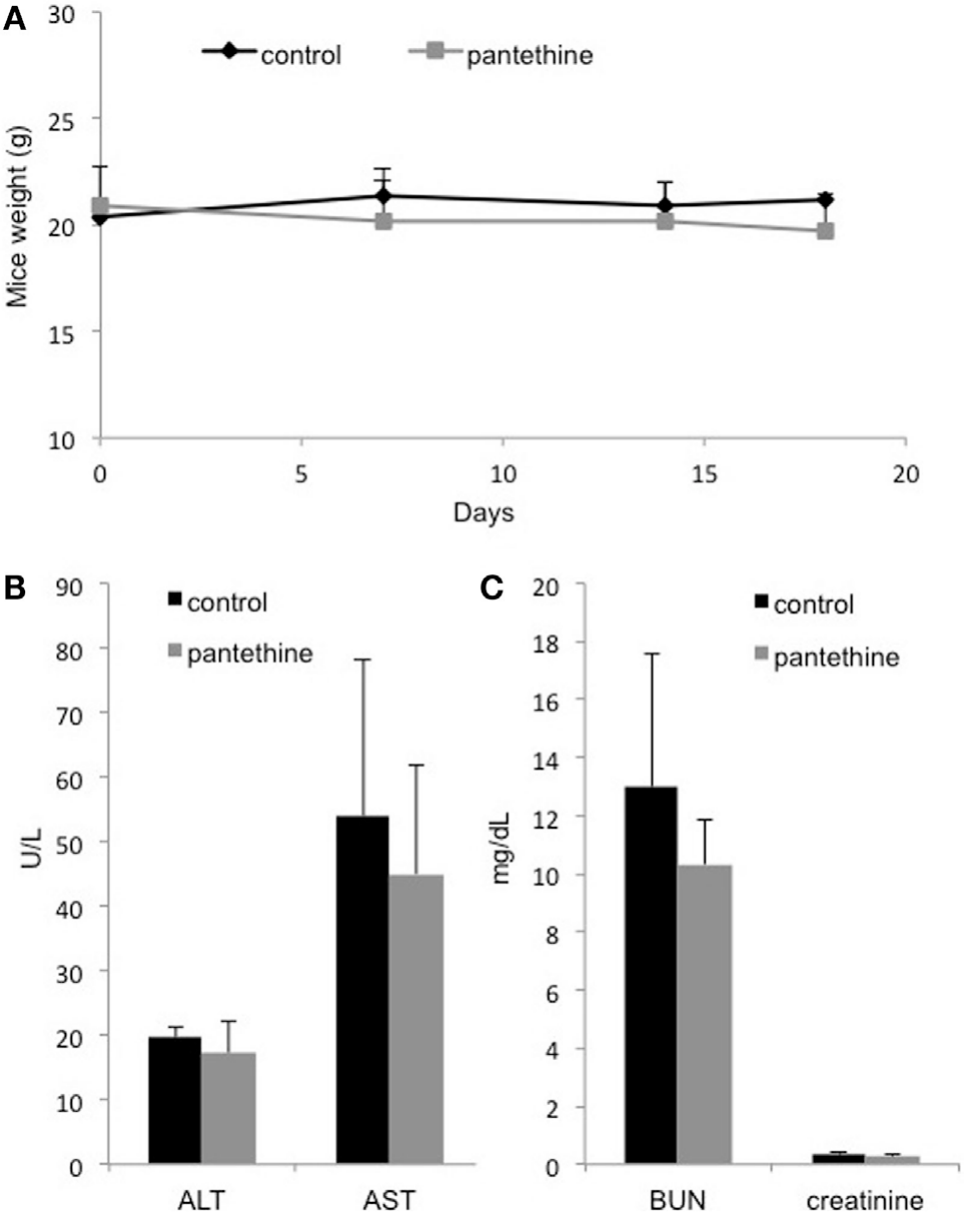
**(A)** Weight curves from control and treated mice. Serum levels of ALT, AST, **(B)** BUN, and creatinine **(C)** in treated and control mice. *n* = 5; mean ± SD are presented.

Renal and hepatic cytotoxicity studies were conducted in MDA-MB-231 tumor-bearing mice. We did not observe any weight loss following 3 weeks of pantethine treatment (Figure 7A). Blood analysis performed at the end of the treatment period revealed neither hepatic (Figure 7B) nor renal toxicity (Figure 7C), as shown by the absence of significant differences in the levels of BUN, creatinine, AST, and ALT between the control mice and the pantethine-treated mice.

## Discussion

The majority of ovarian cancer studies in mouse models use xenografts that are obtained either after subcutaneous implantation (19) or intraperitoneal injection of cancer cells (20). To mimic the tumor microenvironment, more recent models involved direct injection of cells into the ovarian intrabursa (21, 22). Another technique involving the implantation of a preparation of tumor solid pellets into the ovarian bursa was recently described (23). In this model, a pellet was prepared by embedding tumor cells into a collagen matrix to control the number of cells, and to limit their leakage during the injection (23). In the present study, we engrafted a piece of tumor tissue onto the ovary to avoid spilling of cancer cells and to maintain the tumor tissue microenvironment. We have used this technique in the past for prostate cancer (24) and for pancreatic cancer (25). In our model, ascites and metastases in the peritoneal cavity, in the liver, on the diaphragm, and in distal lymph nodes are frequent, similar to human disease, since ovarian carcinoma usually metastasizes along the peritoneum throughout the pelvic and abdominal cavity. In ovarian cancer patients, metastases can be found in lung, skin, pleura, mediastinal, and lymph nodes, and also in bone, brain, or gastrointestinal track (26).

Using our orthotopic model, we identified pantethine as a promising new drug against ovarian cancer, targeting not only tumor progression but also metastases occurrence, and ascites formation. The orthotopic tumor growth could be followed non-invasively using MRI, and we observed slower tumor progression in the treated mice compared to the non-treated ones. While there were no differences in cell proliferation, increased caspase-3 was observed in the treated tumors, linking the tumor growth reduction to an increase in apoptosis. High-resolution ^1^H MRS analysis of tumor extracts revealed a significant decrease of PC and PtCho concentrations in the tumors from treated mice compared to the untreated controls. We used a high dose of pantethine and did not observe any side effects. Pantethine is rapidly eliminated into the urine allowing its administration in humans at a reasonable dose using a slow dispensing device.

Abnormal choline metabolism continues to be identified as one of the most consistent hallmarks of cancer (27). The molecular causes are being gradually unraveled and are providing potential novel targets in the treatment of cancer. Iorio et al. demonstrated that EOC possessed an altered MRS-choline profile, characterized by increased PC content (28). Several studies have demonstrated that targeting choline kinase resulted in a decrease of PC and a reduction of tumor growth (29, 30). Here, we observed a reduction of tumor progression that was associated with a decrease of PC and PtCho.

Several known properties of pantethine may explain these results. A previous study described the inhibition of PtCho synthesis *in vitro* in rat liver microsomal preparations with pantetheine and CoA (31). Here, we observed an *in vivo* effect of pantethine on PtCho level in orthotopically implanted OVCAR3 tumor. Pantethine inhibited fatty acid synthase (FAS), as demonstrated in isolated rat hepatocytes by Bocos and Herrera (32). FAS synthesizes fatty acids using 4′-phospho-pantetheine, which acts as a universal mechanism of transport of intermediates (33–35). Pantethine may inhibit FAS activity through the alteration of the thiol group of the 4′- phospho-pantetheine arm, which covalently carries the pathway intermediates. High FAS activity has been observed in most ovarian cancers and is strongly associated with high aggressiveness and poor patient survival. Inhibition of FAS activity has been shown to be cytotoxic to human cancer cells *in vitro* and *in vivo* (36). Pantethine not only affects cellular fatty acid metabolism but also displays anti-inflammatory properties by maintaining the asymmetric distribution of cell membrane phosphatidylserine, resulting in the inhibition of cellular response to proinflammatory factors (15).

It was recently shown that pantethine affects lipid raft composition causing a decline of the proportion of saturated fatty acid, an increase in mono- and polyunsaturated fatty acid, and a decrease of cholesterol content (17). These changes in raft composition led to an impairment of CXCL12 to bind to its target (17). The CXCL12–CXCR4 axis promotes proliferation, migration, invasion, and metastasis in ovarian cancer (37), therefore its alteration by pantethine may be a mechanism for the reduction of metastases observed in our study.

Finally, it was recently shown that obesity contributes to ovarian cancer metastases formation (38). Adipose tissue is a key component of the ovarian cancer metastatic microenvironment (39, 40). Increased body fat enhances tumor cell–mesothelial cell adhesion and promotes intraperitoneal metastatic dissemination (38). As a known hypolipidemic agent, pantethine may influence metastases formation through its hypolipidemic effects.

In conclusion, pantethine represents a novel potential therapeutic option in patients with ovarian cancer, since it is a well-known and well-tolerated molecule. Further *in vivo* preclinical studies are needed to confirm the beneficial role of pantethine in ovarian cancer and to better understand its mechanism of action.

## Author Contributions

MP: conception and design of the work; acquisition, analysis, and interpretation of data; drafting the work; final approval of the version to be published; and agreement to be accountable for all aspects of the work. BK, FW, and YM: acquisition, analysis, and interpretation of data; final approval of the version to be published; and agreement to be accountable for all aspects of the work. DM, FP, MD, BG, and ZB: design of the work; revising the work; final approval of the version to be published; and agreement to be accountable for all aspects of the work.

## Conflict of Interest Statement

The authors declare that the research was conducted in the absence of any commercial or financial relationships that could be construed as a potential conflict of interest.

## Abbreviations

FAS, fatty acid synthase; IHC, immunohistochemistry; MRI, magnetic resonance imaging; MRS, magnetic resonance spectroscopy; PC, phosphocholine; PtCh, phosphatidylcholine.
