# Supplementary figures and images for: Effect of Pantethine on Ovarian Tumor Progression and Choline Metabolism
Source: Front Oncol. 2016 Nov 16;6:244. doi: 10.3389/fonc.2016.00244 (PMC5110532; doi:10.3389/fonc.2016.00244)

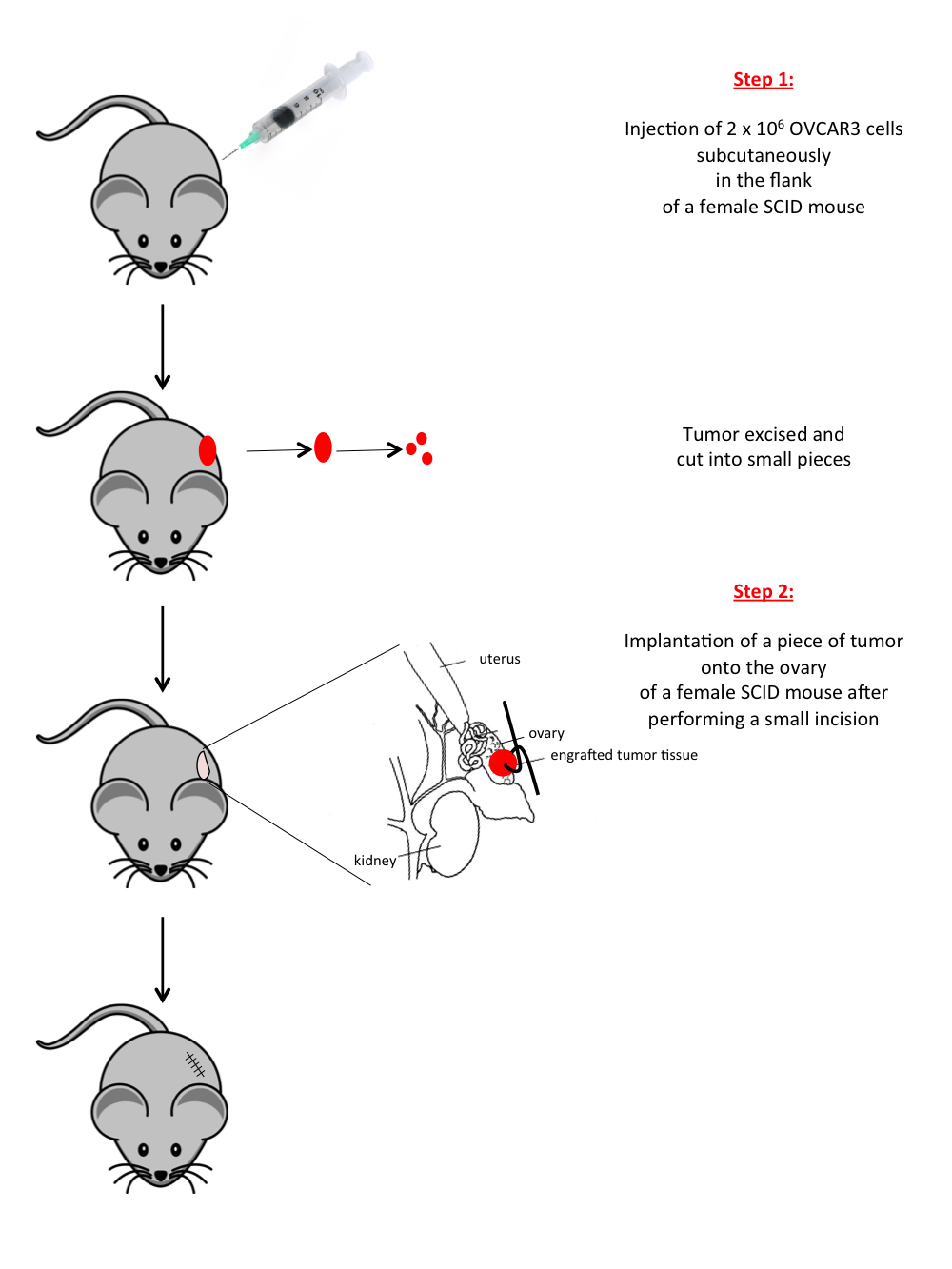

Supplement: Supplementary file 1 [file Image_1.TIFF]
